# Supplementary material for: The power of model-to-crop translation illustrated by reducing seed loss from pod shatter in oilseed rape
Source: Plant Reprod. 2019 Jun 20;32(4):331–40. doi: 10.1007/s00497-019-00374-9 (PMC6820617; doi:10.1007/s00497-019-00374-9)
Supplement: Supplementary file 1 — Supplementary material 1 (PDF 32239 kb) [file 497_2019_374_MOESM1_ESM.pdf]

Online Resource 1

```
HEC           -----MNNYNMNPSLFQNYTWNNIINSSNNNNK--NDDHHHQHNNDPIGMAMDQYTQLHI   53
IND           MMEPQPHHLLMD--------WNKANDLLTQEHAAFLNDPHHLMLDPP-------------   39
BraA.IND.a    MME--HHHLLMN--------WNKPIDLITEENS--FNHNPHFIVDPP-------------   35
BnaA.IND.a    MME--HHHLLMN--------WNKPIDLITEENS--FNHNPHFIVDPP-------------   35
BolC.IND.a    MME--PHHLLMN--------WNKPIDLITQENS--FNHNPHFMVDPP-------------   35
BnaC.IND.a    MME--PHHLLMN--------WNKPIDLITQENS--FNHNPHFMVDPP-------------   35
                    ::  *:        **:  :  .:::    :   *   : *

HEC           FNPFSSSHFPPLSSSLTTTTLLSGDQEDDEDEEEPLEELGAMKEMMYKIAAMQSVDIDPA  113
IND           --PETLIHLD--------------------EDEEYDEDMDAMKEMQYMIAVMQPVDIDPA   77
BraA.IND.a    --SETLSHFQPPPTIFSDHG--GGEEAEEEEEEEGEEEMDPMKKMQYAIAAMQPVDLDPA   91
BnaA.IND.a    --SETLSHFQPPPTIFSDHG--GGEEAEEEEEEEGEEEMDPMKKMQYAIAAMQPVDLDPA   91
BolC.IND.a    --SETLSHFQPPPTVFSDHG--GGEEA---EDEEGEEEMDEMKEMQYAIAAMQPVDIDPA   88
BnaC.IND.a    --SETLSHFQPPPTVFSDHG--GGEEA---EDEEGEEEIDEMKEMQYAIAAMQPVDIDPA   88
                . :  *:                     ::**  *::. **:* * **.**.**:***

HEC           TVKKPKRRNVRISDDPQSVAARHRRERISERIRILQRLVPGGTKMDTASMLDEAIRYVKF  173
IND           TVPKPNRRNVRISDDPQTVVARRRRERISEKIRILKRIVPGGAKMDTASMLDEAIRYTKF  137
BraA.IND.a    TVPKPNRRNVRVSDDPQTVVARRRRERISEKIRILKRMVPGGAKMDTASMLDEAIRYTKF  151
BnaA.IND.a    TVPKPNRRNVRVSDDPQTVVARRRRERISEKIRILKRMVPGGAKMDTASMLDEAIRYTKF  151
BolC.IND.a    TVPKPNRRNVRVSEDPQTVVARRRRERISEKIRILKRMVPGGAKMDTASMLDEAIRYTKF  148
BnaC.IND.a    TVPKPNRRNVRVSEDPQTVVARRRRERISEKIRILKRMVPGGAKMDTASMLDEAIRYTKF  148
              ** **:*****:*:***:*.**.*******.****:*:****:**************.**

HEC           LKRQIR-LLNNNTGYTPPPPQDQASQAVTTSWVSPPPPPSFGRGGRGVGELI          224
IND           LKRQVR-ILQPHSQIGAPMANPSYL-----CYYHNSQP--------------          169
BraA.IND.a    LKRQVR--------LASSASHSAWS-----SYV-------------------          171
BnaA.IND.a    LKRQVRLLLQPHTQLGAPMSDPSCL-----CYYHNSDT--------------          184
BolC.IND.a    LKRQVR-LLQPHTQLGAPMSDPSRL-----CYYHNSDT--------------          180
BnaC.IND.a    LKRQVR-LLQPHTQLGAPMSDPSRL-----CYYHNSDT--------------          180
              ****:*          .. .          .:
```

Online Resource 1. Multiple alignment of IND protein sequences from Arabidopsis and Brassica species including HEC3 from Arabidopsis as an outgroup. Alignment was done using ClustalOmega. Mutated residues used in the analysis of the manuscript are indicated by a black circle.

Online Resource 2

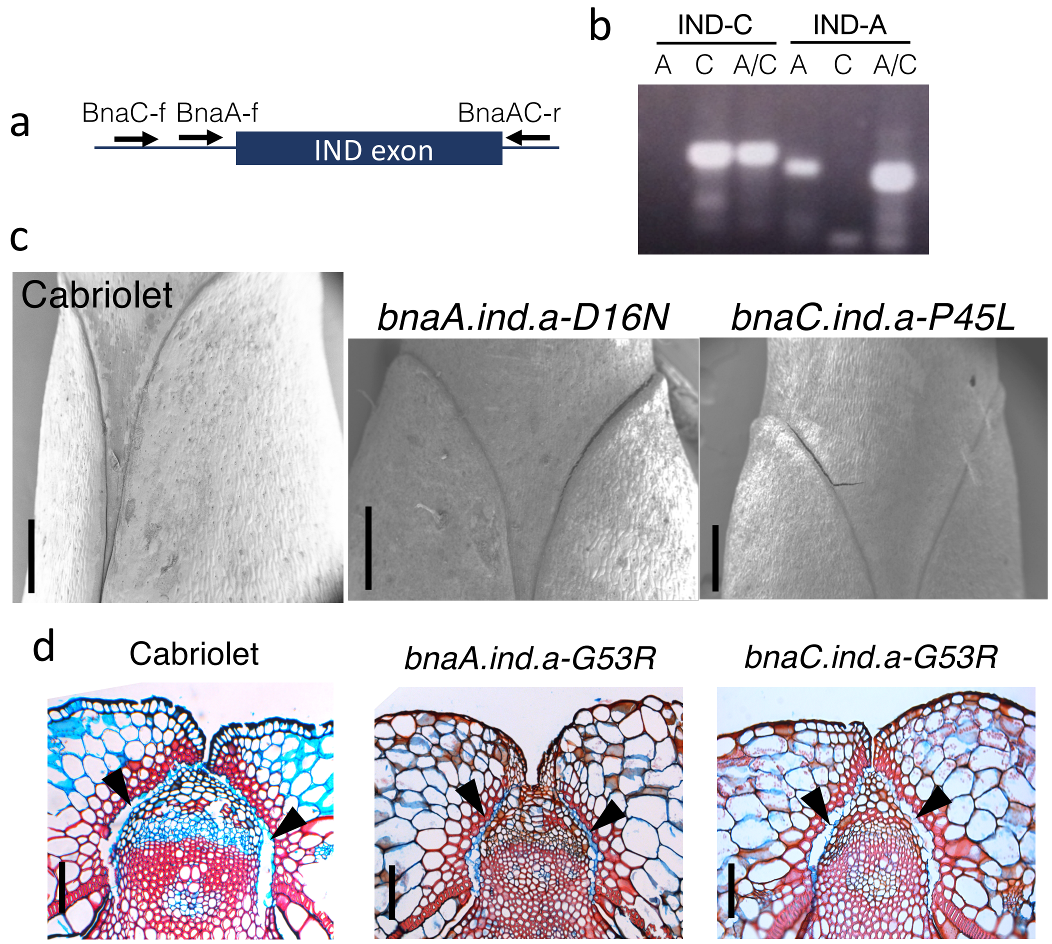

Online Resource 2. a Schematic outline of *IND* gene showing position of paralogue-specific primers (BnaC-f and BnaA-f) and BnaAC-r, which will amplify both. **b** Agarose gel showing paralogue-specific PCR amplification using primers BnaC-f/BnaAC-r to amplify *BnaC.IND.a* (IND-C) or BnaA-f/BnaAC-r to amplify *BnaA.IND.a* (IND-A) on gDNA from *B. rapa* (A), *B. oleracea* (C) and *B. napus* (A/C). **c** Effect of single mutations in *bna.ind* genes. SEM images of mature (stage 17) fruits from Cabriolet (wild type) and indicated *bna.ind* single mutants. **d** Tissue sections of mature (stage 17c) fruits from Cabriolet (wild type) and indicated *bna.ind* single mutants. *Scale bars* correspond to 1 mm in c and 100 µm in d.

Online Resource 3

a

bnaA.ind.a	*bnaC.ind.a* P45L	G53R	R95H	T146I
D16N	X	X	X	X
P45L	-	X	X	-
G53R	X	X	X	X
M70I	X	X	-	X

b

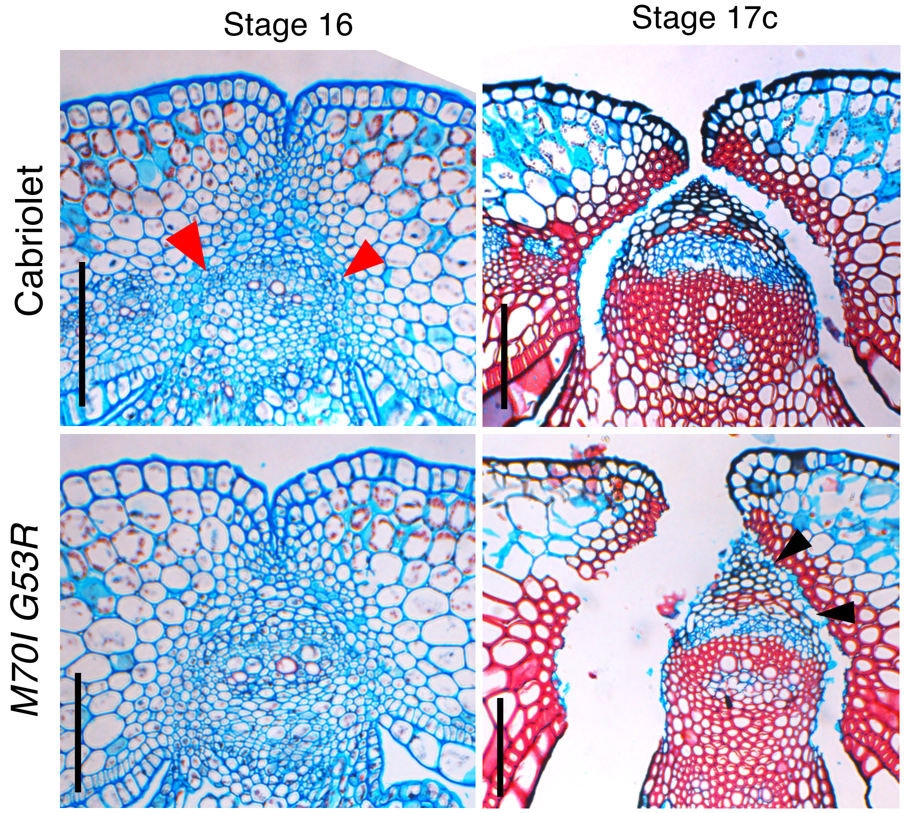

Online Resource 3. a Table of double-mutant combinations. ‘X’ indicates fruits were obtained for RIT while ‘-’ indicates no RIT was performed on those lines due to infertile pods. **b** Tissue sections of fruits from Cabriolet (wild type) and the *bnaA.ind.a-M70I bnaC.ind.a-G53R* (*M70I/G53R*) double mutant at the indicated developmental stages. Red arrowheads point to separation layer primordium cells in Cabriolet. Black arrowheads indicate lack of complete separation in the *M70I/G53R* double mutant. *Scale bars* correspond to 100 µm.

Online Resource 4

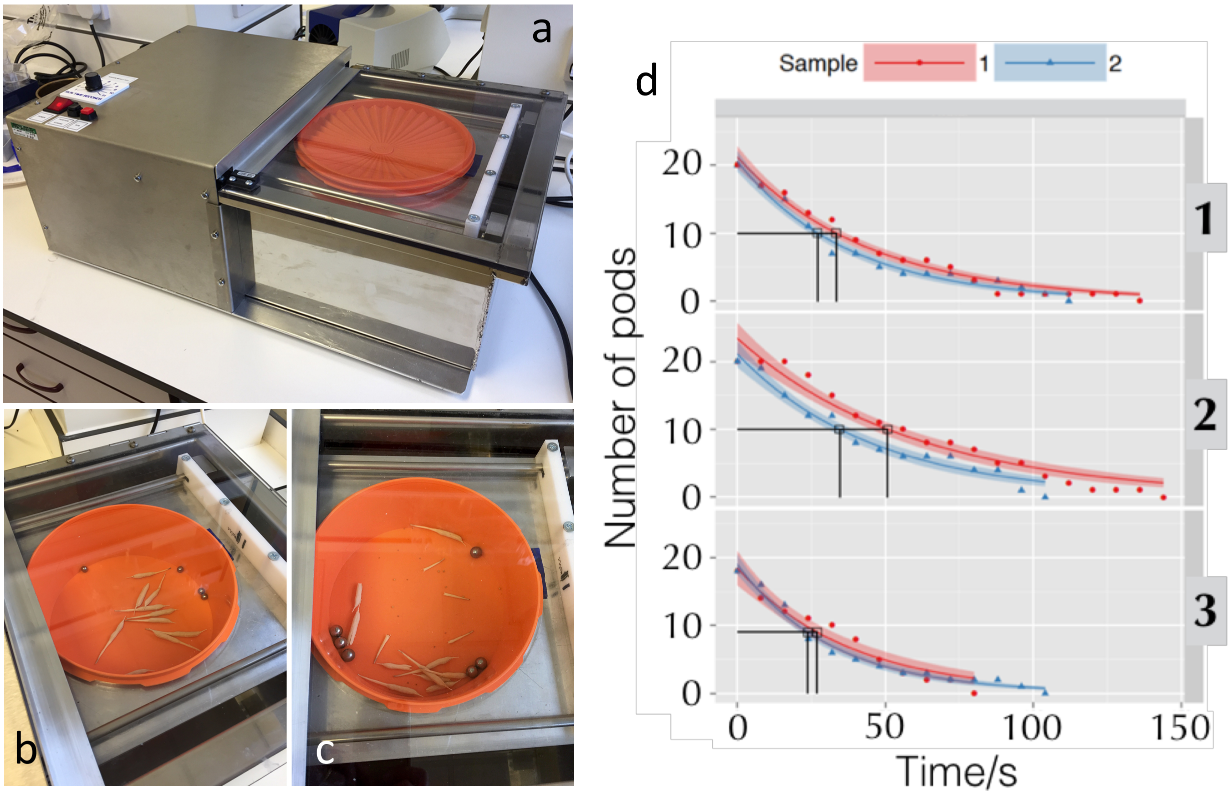

Online Resource 4. Random Impact Test analysis and visualisation of the podshatteR software output. **a** RIT machine on lab bench. **b** photo of sample with ball bearings prior to shaking. **c** photo of sample after one round of 8-sec shaking. **d** example of two samples tested in triplicates with number of remaining intact pods plotted against time of shaking. Values are fitted with decay curves calculated by software generated here. RIT_{50} values are calculated as the time it takes to break 10 pods out of 20.

Online Resource 5

```
AtGA4        MPAMLTDVFRGHPIHLPHSHIPDFTSLRELPDSYKWTPKDDLLFSAAPSPPATGENIPLI   60
BnaC5.GA4    MPTMLTDVFRGHPIHLPHSHQPDFTSLSELPDSYTWTPKDDPLLDAAPSPPAASENIPLI   60
BnaA6.GA4    MPTMLTDVFRGHPIHLPHSHQPDFTSLSELPDSYTWTPKDDPLLDAAPSPPAASENIPLI   60
BnaC8.GA4    MPTVLTDVFRGHPIHLPHTHQPDFTSLSELPDSYTWTSKDDPLFTAPPSPPGAGESIPLI   60
BnaA9.GA4    MPTVLTDVFRGHPIHLPHTHQPDFTSLSELPDSYTWTSKDDPLFTAPPSPPDAGESIPLI   60
             **::**************:* ****** ******.** *** *: * **** :.*.****

AtGA4        DLDHPDATNQIGHACRTWGAFQISNHGVPLGLLQDIEFLTGSLFGLPVQRKLKSARSETG  120
BnaC5.GA4    DLNHPDAANQIGSACRTWGAFQIANHGVPLELLQGIEFLTGSLFQLPVHRKLKAARSETG  120
BnaA6.GA4    DLNHPDAANQIGSACRTWGAFQIANHGVPLELLQGIEFLTGSLFQLPVQRKLKAARSETG  120
BnaC8.GA4    DLNHPDAANQIGRACRTWGAFQIANHGVPLELLQDIEFLTGSLFQLPVQHKLEAARSDAG  120
BnaA9.GA4    DLNHPDAANQIGRACRTWGAFQIANHGVPLELLQGIEFLTGSLFQLPVQRKLKAARSDTG  120
             **:****:**** **********:****** ***.********* ***::**::***::*

AtGA4        VSGYGVARIASFFNKQMWSEGFTITGSPLNDFRKLWPQHHL-NYCDIVEEYEEHMKKLAS  179
BnaC5.GA4    FSGYGVARISSFFNKQMWSEGFTITGSPLNDFRKLWPQHHLNNYCDIVEEYEEQMQKLAS  180
BnaA6.GA4    FSGYGVARISSFFNKQMWSEGFTITGSPLNDFRKLWPQHHLNNYCDIVEEYEEQMQKLAS  180
BnaC8.GA4    FSGYGVARISSFFNKKMWSEGFTITGSPLNDFRKLWPQLHL-NYCDIVEQYEEQMQKLAS  179
BnaA9.GA4    FSGYGVARISSFFNKKMWSEGFTITGSPLNDFRKLWPQLHL-NYCDIVEQYEEQMQKLAS  179
             .********:*****:********************** ** *******:***:*:****

AtGA4        KLMWLALNSLGVSEEDIEWASLSSDLNWAQAALQLNHYPVCPEPDRAMGLAAHTDSTLLT  239
BnaC5.GA4    KLMWLSLTSLGVSEEDIKWASANSDSDWAQSALQLNHYPVCPEPDRAMGLAAHTDSTLLT  240
BnaA6.GA4    KLMWLSLTSLGVSEEDIKWASANPDLNWAQSALQLNHYPVCPEPDRAMGLAAHTDSTLLT  240
BnaC8.GA4    KLMWLSLNSLGVSEEDIKWARVSSDLNWAQSALQLNHYPVCPEPDRAMGLAPHTDSTLLT  239
BnaA9.GA4    KLMWLSLNSLGVTEEDIKWATVSSDLNWAQSALQLNHYPVCPEPDRAMGLAPHTDSTLLT  239
             *****:*.****:****:**  . * :***:******************** ********

AtGA4        ILYQNNTAGLQVFRDDLGWVTVPPFPGSLVVNVGDLFHILSNGLFKSVLHRARVNQTRAR  300
BnaC5.GA4    ILHQNNTAGLQVFRDDLGWVTVPPVPGSLVVNVGDLFHILSNGLFKSVLHRARVNQTRSR  300
BnaA6.GA4    ILHQNNTAGLQVFRDDLGWVTVPPVPDSLVVNVGDLFHILSNGLFKSVLHRARVNQTRSR  300
BnaC8.GA4    ILYQNNTAGLQVFRDDLGWVTVPPVPGSLVVNVGDLFHILSNGLFKSVIHRVRVNQTRPR  300
BnaA9.GA4    ILYQNNTAGLQVFRDDFGWVTVPPVPGSLVVNVGDLFHILSNGLFKSVIHRARVNQTRPR  300
             **:*************:*******.*.*********************:**.****** *

AtGA4        LSVAFLWGPQSDIKISPVPKLVSPVESPLYQSVTWKEYLRTKATHFNKALSMIRNHREE   358
BnaC5.GA4    LSVAFLWGPQSDIKISPVPKLVSPVGSPLYRSVTWTEYLRTKATHFNEALSMIRNHIDE   359
BnaA6.GA4    LSVAFLWGPQSDIKISPVPKLVSPVGSPLYRSVTWTEYLRTKATHFNEALSMIKNDIDE   359
BnaC8.GA4    LSVAFLWGPRSDTKISPVPKLVSPDESPLYRSVTWTEYLRTKATHFNKALSMIRNHREK   358
BnaA9.GA4    LSVAFLWGPRSDTKISPVPKLVSPDESPLYRSVTWTEYLRTKATHFNKALSMIRNHREK   358
             *********:** ***********  ****:****.***********:*****:*. ::
```

Online Resource 5. Multiple alignment of GA4 protein sequences from Arabidopsis and Brassica species. Alignment was done using ClustalOmega. Mutated residues used in the analysis of the manuscript are indicated by a black circle.

Online Resource 6

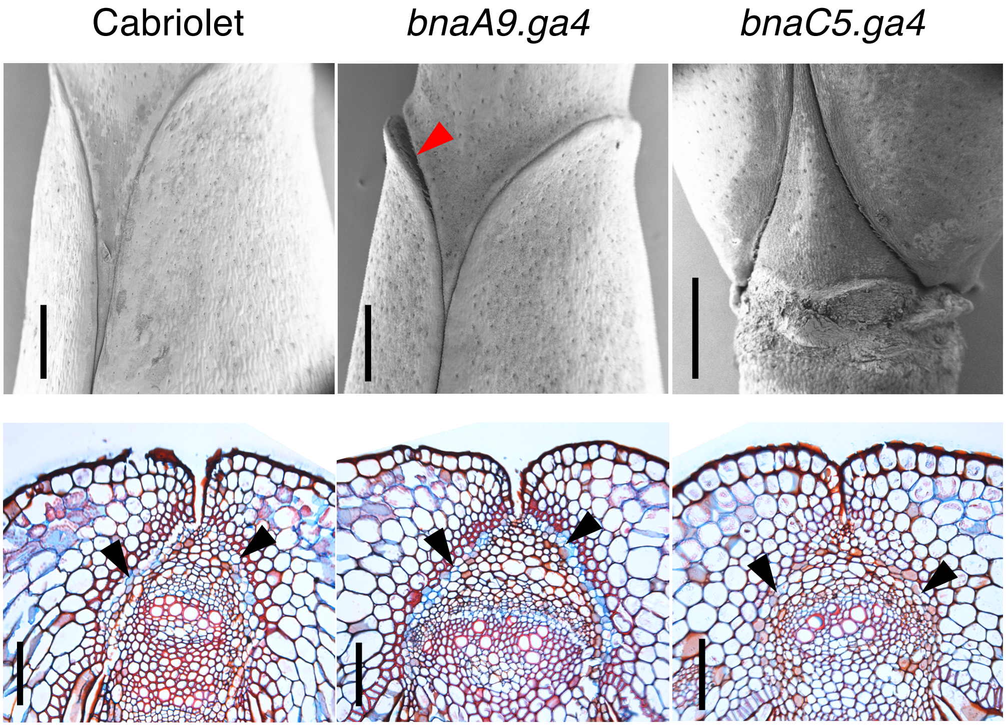

Online Resource 6. Effect of mutations in *bna.ga4* genes. SEM (upper row) and tissue section (lower row) images of mature (stage 17) fruits from Cabriolet (wild type), *bnaA.ga4* and *bnaC.ga4* single mutants. Red arrowhead indicates valve that is opening, black arrowheads point to separation layer in cross sections. *Scale bars* correspond to 1 mm for SEMs and 100 µm for sections.

Online Resource 7

Oligonucleotides used in this study

Bna.GA4

A6-F	gtccaacacttccataatcttcc
A6-R	GAATGACATGGCGAAATCTCTGT
C5-F	gactctccgactcttccataatt
C5-R	ACATGGCAAATTTTCGGTTTCT
C8-F	gacacaaaacatctatcgaat
C8-R	gccgtctttagcatatgtaaattgagccg
A9-F	ccaaaacaagagcttaaacatatgc
A9-R	gcaaccaatatatatcatgacc

Bna.IND

IND A-F	aacattcatacacgcactac
IND A-R	atcaacatgaaacgcgtgat
IND C-F	ccgcaaatacaaacatatttagt
IND C-R	atcaacatgaaacgcgtgat
