# Supplementary material for: The power of model-to-crop translation illustrated by reducing seed loss from pod shatter in oilseed rape
Source: Plant Reprod. 2019 Jun 20;32(4):331–40. doi: 10.1007/s00497-019-00374-9 (PMC6820617; doi:10.1007/s00497-019-00374-9)
Supplement: Supplementary file 2 — Supplementary material 2 (DOCX 1634 kb) [file 497_2019_374_MOESM2_ESM.docx]

**Online Resources**

**The podshatteR software**

podshatteR is a browser tool or web app for plotting and analysing pod shatter data collected from Random Impact Tests (RIT) assays (see Materials and Methods). This app works for data that may have repeated samples and/or used a randomised block design.

It facilitates visualisation of measured pod shatter RIT data and determine the ‘half-life’ (RIT_50_) of each sample using a curve fitting routine in R.

podshatteR is available at https://github.com/nstjhp/podshatteR

**Generation of podshatterR software**

podshatteR is released as a collection of R scripts and interacted within a browser through the shiny package. Launch the web app with the command

R -e "shiny::runApp(’~/shinyAppFolderLocation’)"

After launching the web browser and navigating to the correct port you will see the application landing page which explains the details of how to use the tool. On the first tab the tool can read in your input RIT data formatted as described on the homepage in a csv file. A plot of the data is produced to allow visual inspection of any errors or ambiguities that can then be resolved before proceeding. The following tab calculates the fit to the data. The model used is an exponential decay function to keep the number of free parameters minimal i.e.

*Value* = *Aexp*(-*b* × *Time*)

where A is the intercept (which should be similar to the starting number of pods), and b is the decay rate. Through propagation of errors confidence intervals are computed for each sample. The next tab allows for the plotting of the half-life locations and the ability to download a csv file containing all the calculated half-lives.

The half-life is calculated for each sample as the value at which half the initial number of pods has shattered, following the model fit. In the final tab you can download a subset of the plots in PDF format. The x-axis can be fixed across the rows/columns, or can be fixed, and the final plot size of the downloaded PDF can be chosen by the user.

The podshatteR code was built with R version 3.1.2 and uses the following packages (versions):

- shiny (0.11.1)

- ggplot2 (1.0.0)

- grid (3.1.2)

- reshape2 (1.4.1)

- plyr 1.8.1

- minpack.lm (1.1-8)

- propagate (1.0-4)

**Using the podshatteR software**

In order to use podshatteR, a desktop PC equipped with Statistical package R (version 3.2 or later) is required. The app is written in R using the `shiny` package for display in the browser and was built using the following versions:

- `R` v3.1.2

- `shiny` v0.11.1

- `ggplot2` v1.0.0

- `grid` v3.1.2

- `reshape2` v1.4.1

- `plyr` v1.8.1

- `minpack.lm` v1.1-8

- `propagate` v1.0-4

Step-by-step procedure

1. After the installation of R, the following packages will be required to run the App. Type (or copy from the INSTALL file within the podshatteR folder) the command below, to install the required packages:

install.packages(c("shiny", "ggplot2", "grid", "reshape2", "plyr", "minpack.lm", "propagate"), dependencies=TRUE)

2. If running for the first time, restart R, then type the command to load the Shiny R package:

>library(shiny)

3. In the File dropdown menu, change the working directory to the podshatteR folder. To open and run the application in a web browser, type the command:

>runApp()

The web app is split up into a number of sections, which can be navigated using the bar at the top.

4. Select the Upload and plot data tab

- Click `Browse...` to find and upload data. This will have to be saved as a .CSV file in the required format (shown on the About tab). Please note capital letters are required at the start of each column header, failure to provide this will prevent data from being loaded.

- This tab will then read the data, display them in a table, and plot the data with lines to facilitate spotting of errors in the data entry.

- The plots show every line with repeated samples in different colours and symbols.

If using more than one block, then the separate blocks will be shown in separate sub-tabs.


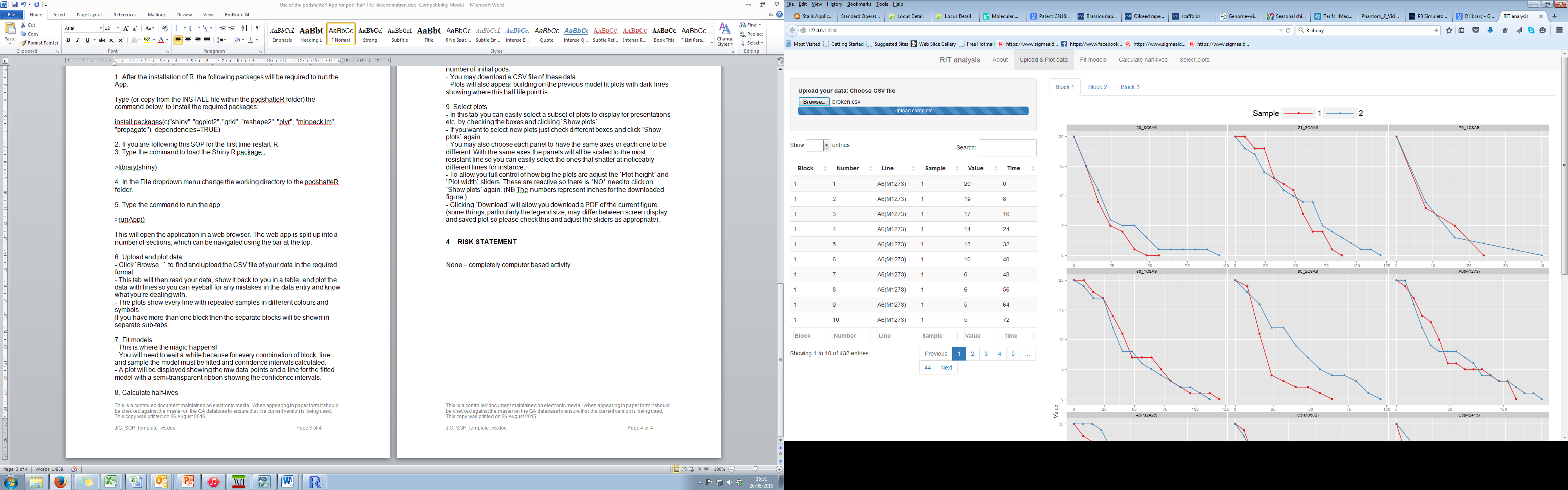


Fig.1 An example of data plotted in the upload and plot data tab.

5. Select the Fit models tab and press the Fit models button. For every combination of block, line and sample the model must be fitted and confidence intervals calculated. A plot will be displayed showing the raw data points and a line for the fitted model with a semi-transparent ribbon showing the confidence intervals.


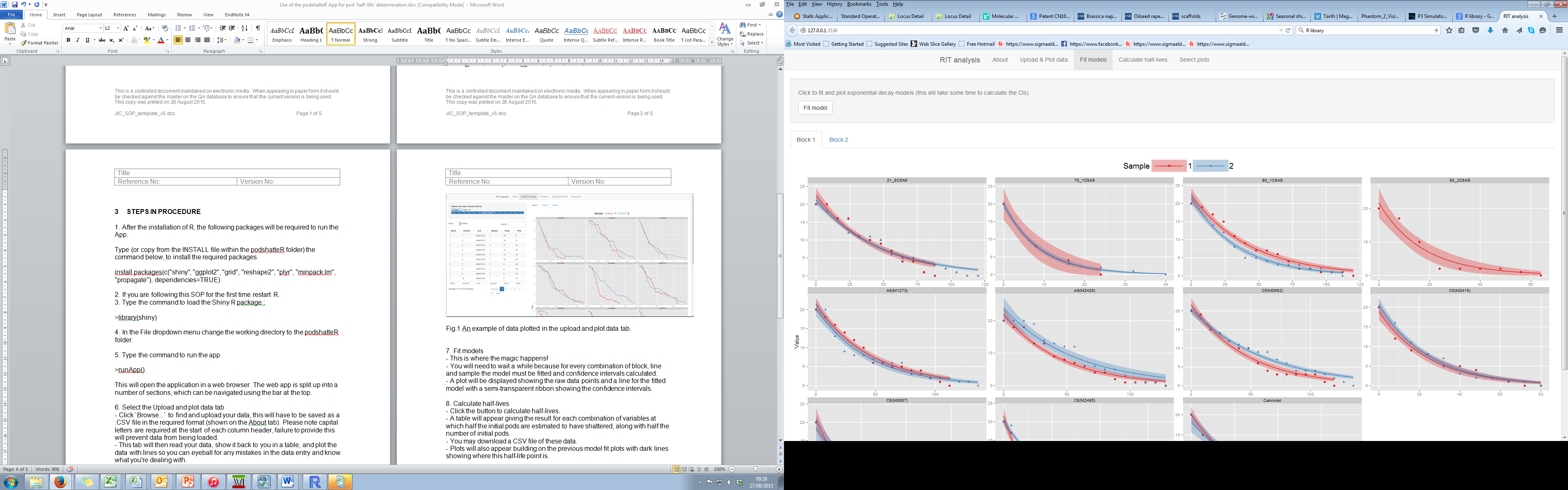


Fig 2. An example of the Fit models tab showing plotted curves and confidence intervals

6. Select the Calculate half-lives and click the button. A table will appear giving the result for each combination of variables at which half the initial pods are estimated to have shattered, along with half the number of initial pods. Plots will also appear building on the previous model fit plots with dark lines showing where this half-life point is. A .CSV file of these data can be downloaded using the download button.


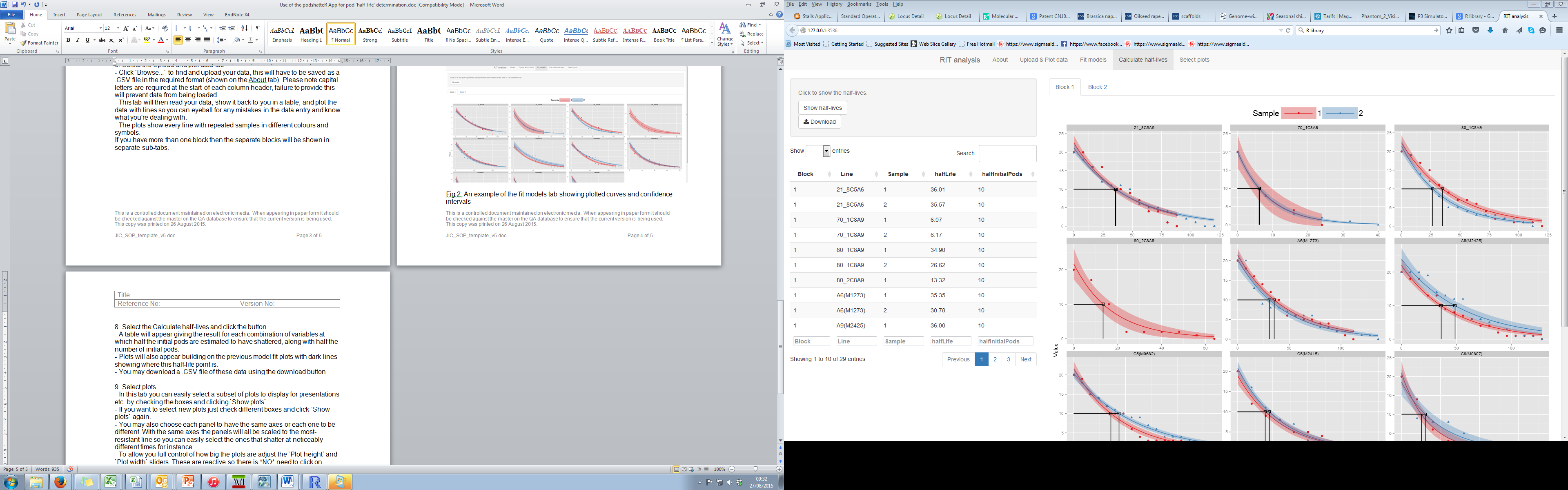


Fig. 3 The output from the Calculate half-lives tab showing half-life estimate table (left) and half-life plotted on the fitted curves (right).

7. Select plots tab allows simple selection of a subset of plots to display for presentations etc. by checking the boxes and clicking `Show plots`. If new plots are selected, simply check different boxes and click `Show plots` again.

Each panel can be chosen to have the same axes or each one to be different. With the same axes the panels will all be scaled to the most-resistant line so you can easily select the ones that shatter at noticeably different times for instance.

To allow full control of plot size, adjust the `Plot height` and `Plot width` sliders. These are reactive so there is no need to click on `Show plots` again (numbers represent inches for the downloaded figure).

Clicking `Download` will facilitate downloading of a PDF of the current figure (some aspects, particularly the legend size, may differ between screen display and saved plot, but this can be adjusted using the sliders).
